# Ischemic proctitis caused by a superior rectal arteriovenous fistula: a case report and literature review

**DOI:** 10.3389/fgstr.2025.1700403

**Published:** 2026-01-02

**Authors:** Jiaqi Dong, Ming Zhao, Fengju Yuan, Ni Huang

**Affiliations:** 1 Department of Gastroenterology, Deyang People’s Hospital, Affiliated Hospital of Chengdu University of Traditional Chinese Medicine, Deyang, Sichuan, China; 2 Department of Pathology, Deyang People’s Hospital, Affiliated Hospital of Chengdu University of Traditional Chinese Medicine, Deyang, Sichuan, China; 3 Department of Radiology, Deyang People’s Hospital, Affiliated Hospital of Chengdu University of Traditional Chinese Medicine, Deyang, Sichuan, China

**Keywords:** ischemic proctitis, hemoproctia, rectal stricture, superior rectal arteriovenous fistula, laparoscopic anterior resection of rectum, embolization

## Abstract

Ischemic proctitis is a rare but severe condition characterized by ischemic injury to the rectum due to insufficient blood supply from the vessels feeding the rectum. Due to the rectum’s rich collateral circulation, ischemic proctitis is uncommon. We present a case of a 61-year-old man with ischemic proctitis presenting primarily with rectal bleeding. Angiography confirmed the presence of a superior rectal arteriovenous fistula. The diagnostic process was quite challenging. The patient underwent endoscopic hemostasis and interventional embolization, and eventually underwent proctectomy due to rectal stenosis. A literature review on ischemic proctitis is also included.

## Introduction

Ischemic proctitis (IP) is a rare condition, accounting for only 2%–5% of all ischemic colitis cases (1). This localized rectal lesion typically results from severe vascular disorders or acute vascular occlusion (2, 3). The rectum is less prone to ischemia due to its rich blood supply and collateral circulation, which include the superior rectal artery (a branch of the inferior mesenteric artery), middle rectal artery (a branch of the internal iliac artery), and inferior rectal artery (a branch of the internal iliac artery). However, ischemic proctitis occurs when the rectal blood supply is compromised (4). Herein, we report a rare case of ischemic proctitis caused by a superior rectal arteriovenous fistula.

## Case report

A 61-year-old male presented with tenesmus and mucinous bloody stools without abdominal pain, distension, nausea, vomiting, or fever. Physical examination revealed no significant abdominal findings. He had a 3-year history of hypertension and hyperlipidemia, managed with amlodipine and rosuvastatin. There was no history of abdominal surgery or trauma. The patient was a 30-year smoker and consumed alcohol socially.

**Figure 1:**
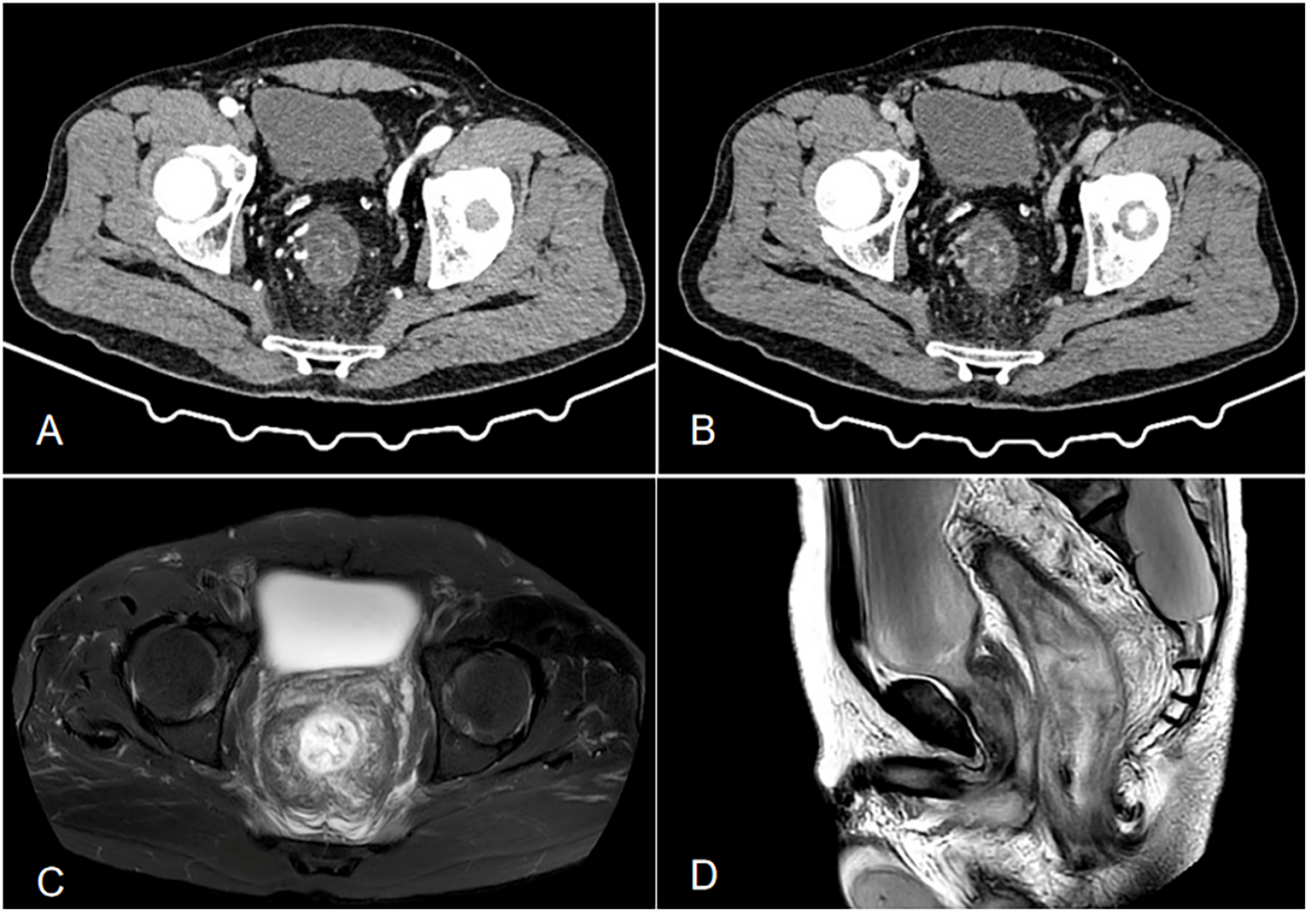
**(A, B)** Contrast-enhanced CT shows local wall thickening and heterogeneous enhancement of the sigmoid colon, with increased and thickened surrounding blood vessels, dilation and effusion of the proximal intestinal lumen, thickening of the distal rectosigmoid colon wall showing a layered change, and layered enhancement after enhancement. Its feeding artery is thickened, with exudation and fascia thickening seen around(a is the venous phase, and b is the arterial phase). **(C, D)** Enhanced pelvic MRI demonstrated segmental wall thickening of the rectum and sigmoid colon with marked blurring in the perirectal fat plane. (c is T2-weighted fat-suppressed sequence, and is Sagittal T2WI sequence).

**Figure 2:**
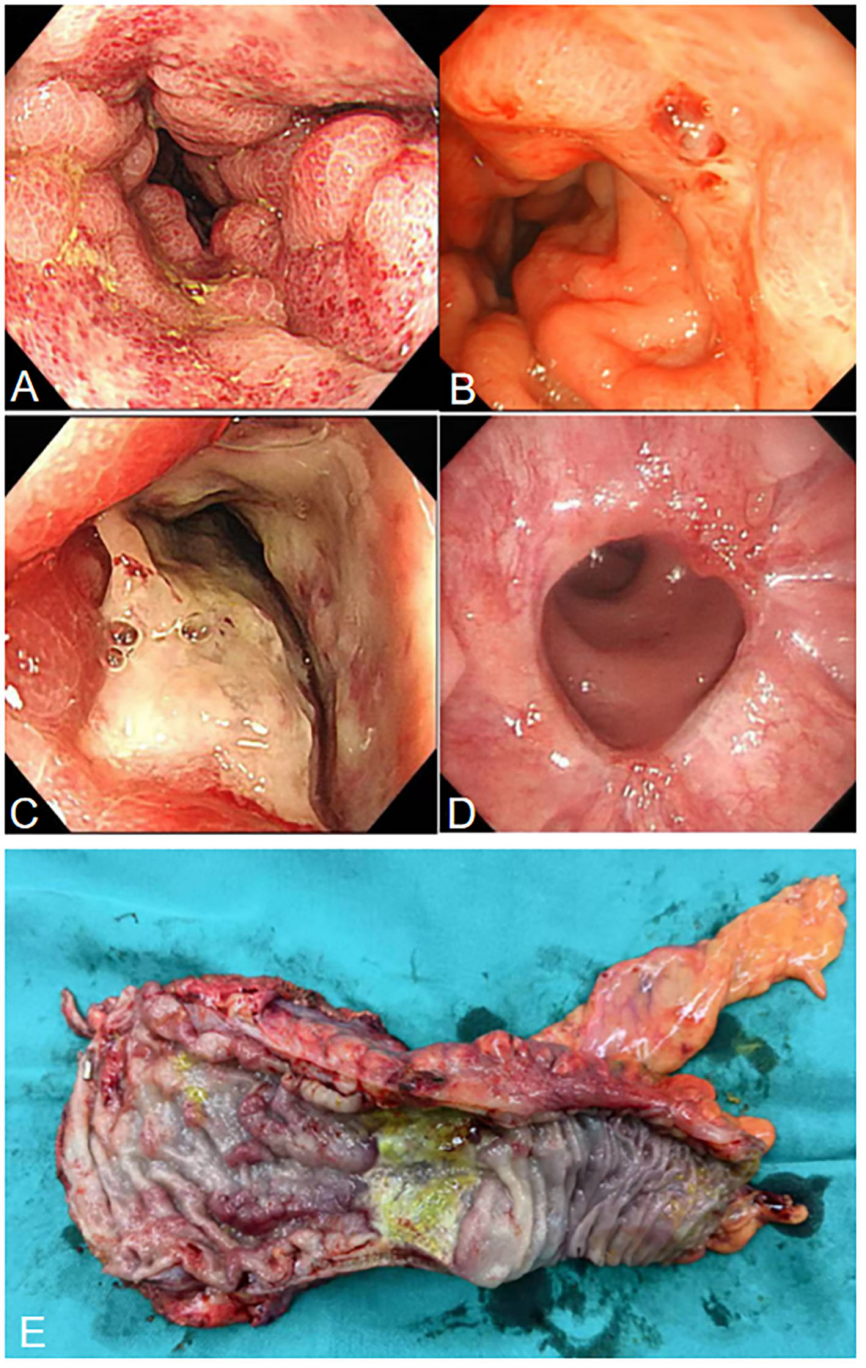
**(A)** Colonoscopy shows hyperemia, edema, and erosion of the rectal mucosa. **(B)** Colonoscopy shows a red thrombus head is visible in the rectum, approximately 3 cm from the anus. **(C)** Colonoscopy shows ulcers and strictures at the rectosigmoid junction. **(D)** Colonoscopy shows that the rectal anastomosis is healing well. **(E)** Surgical specimen showing ischemic ulcers and stricture in the rectum.

An initial blood test revealed white blood cell count (13.69×10^9^/L) with 84.1% neutrophils. Liver function, renal function, coagulation, tumor markers, erythrocyte sedimentation rate, and autoantibodies were normal. Infectious workup (c-ANCA, p-ANCA, EBV, CMV, hepatitis viruses, syphilis, HIV) was negative. Fecal cultures and Clostridioides difficile tests were repeatedly negative. Contrast-enhanced abdominal CT (**Figure 1**) showed rectosigmoid wall thickening with submucosal edema, increased mesorectal vascularity, and partial large bowel obstruction. Enhanced pelvic MRI demonstrated segmental wall thickening of the rectum and sigmoid colon with marked blurring in the perirectal fat plane. Colonoscopy (**Figure 2A**) revealed mucosal edema and erythema from the rectum to the segment 20 cm from the anus, with the remaining colonic mucosa being normal. Pathological examination revealed chronic inflammation of the rectal mucosa with erosion, which is considered to be ulcerative proctitis. Mesalazine, steroid therapy and antibiotics were initially administered as treatment.

**Figure 3:**
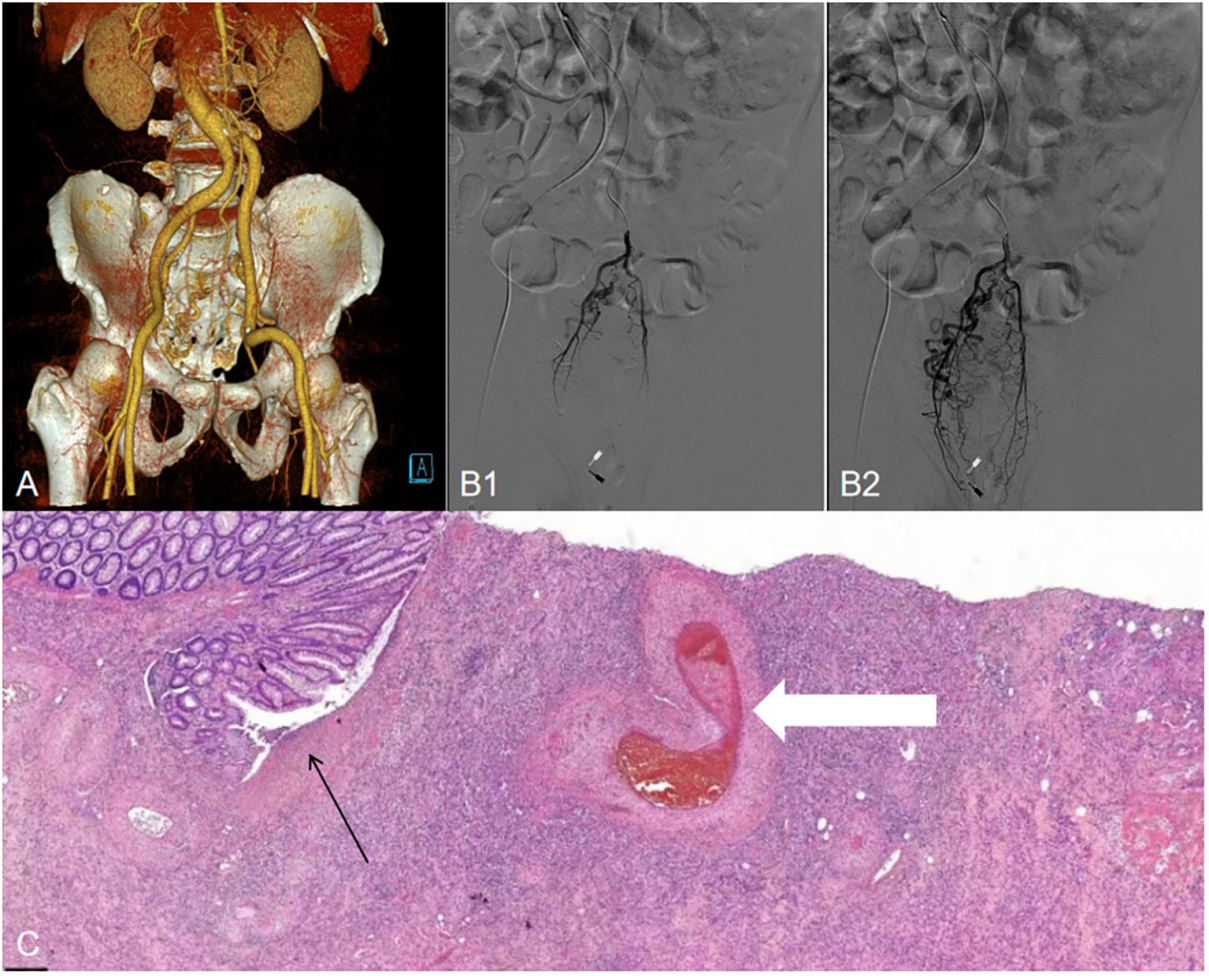
**(A)** Three-dimensional reconstructed CT shows a thickened, tortuous vessel shadow accompanying the superior rectal artery in the arterial phase, with enhancement degree consistent with that of the surrounding veins in the venous phase. **(B1, B2)**, Angiogram demonstrating a superior rectal artery AVF with early venous shunting. **(C)** Histopathological examination (H&E staining, magnification×10) shows intestinal mucosal erosion and necrosis (black arrows), along with vascular wall thickening and thrombosis (white arrows).

After two weeks of treatment, the patient suddenly developed massive hematochezia. Despite administration of somatostatin for hemostasis, bleeding persisted. Repeat blood tests revealed hemoglobin dropped to 66 g/L, prompting immediate blood transfusion and supportive care. Emergency colonoscopy (**Figure 2B**) revealed a large amount of blood in the rectal lumen upon insertion, with multiple rectal ulcers accompanied by mucosal erosion and bleeding. A red thrombus head was observed approximately 3 cm from the anus, which was then clipped with titanium clips. Pathological examination showed deposition of fibrinous necrotic material in the vascular lumen, leading to a diagnosis of ischemic proctitis (IP). Therefore, mesalazine was discontinued, and papaverine was added to dilate blood vessels. During a multidisciplinary discussion, the patient’s CT scans (**Figure 3A**) were re-reviewed, which showed a thickened and tortuous vessel shadow accompanying the superior rectal artery in the arterial phase, with enhancement degree consistent with that of surrounding veins in the venous phase, suggesting the possibility of an arteriovenous fistula. Further angiography (**Figure 3B**) demonstrated a tortuous, enlarged right superior rectal artery with early filling of vein, confirming an arteriovenous fistula (AVF). Transcatheter coil embolization of the AVF was performed. However, due to the large size of the AVF, only partial embolization could be performed.

Within one month after the operation, the patient was on a liquid diet, with no abdominal pain or hematochezia, and occasionally passed a small amount of mucous stool. Follow-up colonoscopy (**Figure 2C**) revealed swelling of the rectal mucosa, gradual luminal stenosis with the formation of a large ulcer, which prevented the endoscope from passing through. No signs of malignant tumors were detected in the biopsy. Finally, he was transferred to the surgery department for laparoscopic anterior resection of the rectum (Dixon operation) and diverting ileostomy of the terminal ileum (**Figure 2E**). Postoperative pathology confirmed ischemic IP (**Figure 3C**). Follow-up colonoscopy (**Figure 2D**) 4 months later revealed normal colonic mucosa.

## Discussion

IP typically occurs in elderly patients with severe vascular diseases, low-flow states, or after aortic/iliac artery surgery (3, 5, 6). Other etiologies include radiation therapy, vasculitis, mesenteric venous myointimal hyperplasia, systemic lupus erythematosus, and anaphylactic shock (1, 7, 8). Common symptoms of IP include hematochezia, lower abdominal pain, anal pain, diarrhea, and tenesmus, with systemic symptoms (fever, nausea, vomiting) in severe cases. These nonspecific symptoms overlap with inflammatory bowel disease, infectious colitis, and colorectal cancer. Serological and stool tests exclude infectious causes (4), while colonoscopy and histology confirm the diagnosis (9).In addition, colonoscopy allows for direct visualization of mucosal lesions, as well as assessment of their severity and extent.

Similar to ischemic colitis, IP severity ranges from superficial ischemia to full-thickness necrosis and perforation (10, 11). Acute-phase mucosal edema and hemorrhage are reversible, but prolonged ischemia leads to mucosal necrosis, ulceration, and risk of transmural necrosis with gangrene or perforation. Chronic progression may cause fibrosis and rectal stricture (5, 12). This case clearly shows that the rectal mucosa progressed from congestion and edema to ulceration, bleeding, and then to rectal stenosis, with a rapid progression of the condition.

Treatment depends on severity and etiology: conservative therapy (broad-spectrum antibiotics, fluid resuscitation) for mild cases, and rectectomy for severe cases with necrosis, perforation, or major hemorrhage (13). Transmural necrosis is associated with a 40% mortality rate (14), highlighting the need for early identification to prevent complications (15).

In this report, we describe a case of ischemic proctitis (IP) secondary to a superior rectal AVF. To date, only 3 cases of ischemic proctitis associated with arteriovenous fistula have been reported (16–18). AVF, a rare vascular anomaly, allows direct arterial-to-venous shunting without capillary filtration, either congenital or acquired (19, 20). Our patient had no laparotomy history, and prior colonoscopy revealed sigmoid varices, suggesting a congenital superior rectal AVF (initial images unavailable). The proposed mechanism was reduced distal rectal perfusion from arterial shunting into veins, combined with elevated venous pressure impairing return flow leading to ischemic proctitis. Although interventional embolization was attempted, partial success occurred due to a large fistula. Even with only partial success, vascular embolization is indeed a relatively safe and less invasive therapeutic measure for managing the risk of lower gastrointestinal bleeding (21), as demonstrated in this patient case.

Rectal stenosis secondary to rectal ischemia is relatively rare due to the large lumen of the rectum. However, in this case, the patient developed rectal stenosis caused by circumferential cicatricial ulcers, and thus ultimately underwent surgical treatment.

## Conclusion

Ischemic proctitis is rare but severe, with colonoscopy, radiology, and clinical features key to diagnosis. Early diagnosis and timely treatment are crucial for improving patient prognosis. For severe stricture cases unresponsive to treatment, proctectomy may be required. Despite the extremely low incidence of this disease, clinicians should remain vigilant about it and include ischemic proctitis in the differential diagnosis of lower gastrointestinal bleeding.

## Data Availability

The raw data supporting the conclusions of this article will be made available by the authors, without undue reservation.
